# HIDSAG: Hyperspectral Image Database for Supervised Analysis in Geometallurgy

**DOI:** 10.1038/s41597-023-02061-x

**Published:** 2023-03-23

**Authors:** Alejandro Ehrenfeld, Álvaro F. Egaña, Felipe Santibañez-Leal, Felipe Garrido, Marcia Ojeda, Brian Townley, Felipe Navarro

**Affiliations:** 1 Advanced Laboratory for Geostatistical Supercomputing (ALGES), Advanced Mining Technology Center (AMTC) - Department of Mining Engineering, University of Chile, Santiago, 8370451 Chile; 2 Advanced Mining Technology Center (AMTC) - Department of Mining Engineering, University of Chile, Santiago, 8370451 Chile

**Keywords:** Scientific data, Computer science, Mineralogy

## Abstract

Supervised analysis using spectral data requires a well-informed characterisation of the response variables and abundant spectral data points. The presented hyperspectral dataset comes from five sets of geometallurgical samples, each characterised by different methods. To provide the spectral data, all mineral samples were scanned with SPECIM VNIR and SWIR hyperspectral cameras. For each subset the following data are provided 1) hyperspectral reflectance images in the VNIR spectral range (400–1000 nm wavelength); 2) hyperspectral reflectance images in the SWIR spectral range (900–2500 nm wavelength); 3) hyperspectral reflectance images in the VNIR-SWIR range (merged to SWIR spatial resolution); 4) RGB images constructed from hyperspectral data using a Bilateral Filter based sensor fusion method; 5) response variables representing mineral sample characterisation results, provided as training and validation data. This dataset is intended for use in general regression and classification research and experiments. All subsets were validated using machine learning models with satisfactory results.

## Background & Summary

This data set has been constructed over a period of seven years of applied research carried out with mining industry partners.

The mineral samples selected for this work and the characterization methods used to generate their response variables represent some of the main types of information that are considered when modeling copper-bearing mineral deposits, and planning and controlling their milling-flotation metallurgical processes^1^.

The delivered values of response variables consist of geochemical, mineralogical, and metallurgical data obtained from laboratory and sensor analyses of mineral samples taken from both drill core composites and milling feeding lines.

Multipixel hyperspectral imagenology was used to capture spectral data, within the 400 to 2500 nm wavelength range. Tens of thousands of spectra pixels from each mineral sample were registered, providing statistically representative spectral information from their surface. As a result, the presented dataset is useful for any supervised or unsupervised analysis methodology, not necessarily related to geometallurgical aspects.

Ore material destined for metallurgical processes corresponds to a complex mixture of minerals. Each ore rock type coming from the mining extraction process is part of a specific ore body and has resulted from a sequence of ore deposits forming geological processes.

The samples of the dataset presented belong to mineralized ore rocks of copper and copper-molybdenum porphyry deposits. These kinds of rocks have undergone several transformations due to magmatic and hydrothermal phenomena and their mineralogical composition varies along different geometallurgical units of each ore body. Besides, once the ore is crushed and stocked for milling, the mixture is even more complex.

The spectral data presented has inherent stochastic characteristics. Even if a mineral sample has been homogenized, each pixel of a hyperspectral image is different. On the other hand, the interest variables can be represented by a statistical distribution of all pixels corresponding to a particular sample. There are no representative spectra for one mineral sample or pure minerals as in the case of the dataset presented by Fasnach *et al*.^2^.

Five subsets of data are presented, each one belonging to a different ore body and counting with different characterization processes as labeling for machine learning supervised experiments, as well as for clustering. No excessive attention is paid to the spectral data itself or its accuracy, as this dataset is not intended for use in feature extraction or pattern recognition algorithms.

The main focus of this dataset is Machine Learning algorithms, whose main purpose is to estimate the joint probability distribution between a response variable and a set of predictor variables (the spectra in our case) while minimizing, for example, the empirical risk associated with the chosen model^3^. In this regard, special attention should be paid to the complexity of the model and to maintaining statistical consistency between those variables (response and predictor) in order to: a) avoid over-fitting the data and b) facilitate the generalisability of the model^4^. In other words, when data has become too polished - i.e. too accurate - in the sense of pattern recognition, there is a danger that it loses generalisability in a Machine Learning model. To this end, the response variables coming from standardized chemical, mineralogical or metallurgical characterization for the ore samples have a consistent relationship with spectral data produced for each sample set.

Each of the five spectral data subsets has been acquired with constant acquisition parameters, such as binning, frame rate, sample movement speed, focus, and integration time. However, the environmental conditions of the acquisition process have only been partially controlled to provide variability in the spectral data in order to test robust estimation models that can then be used in real-world situations.

## Methods

### Mineral sample sets and sensing data description

**Fig. 1:**
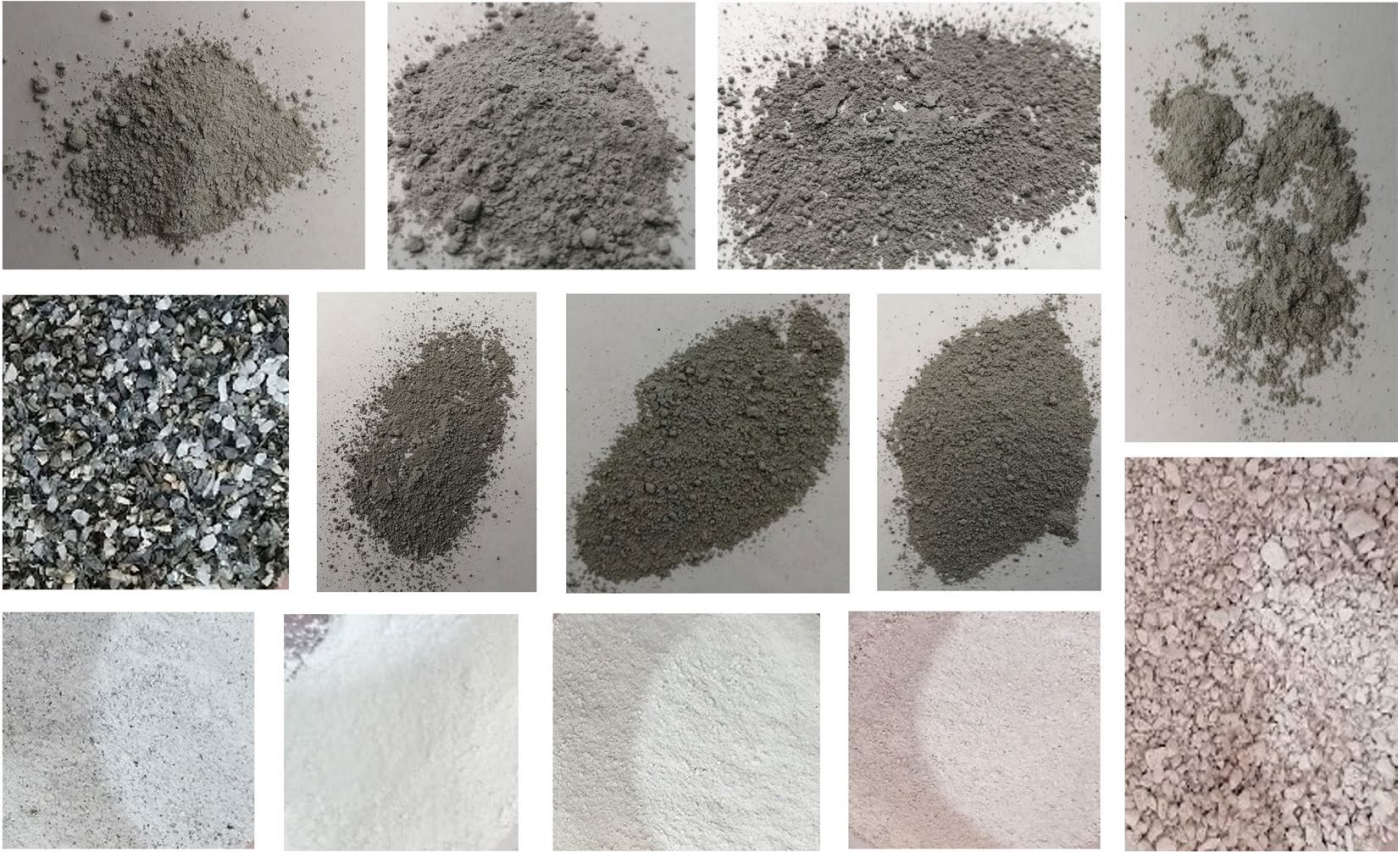
RGB images of some selected mineral samples. The four samples above and the three in the centre that are fine powder have an actual size of approximately 70 by 70 mm. The two samples where separate grains are recognisable have an average grain size of 3.5 mm and the four fine powder samples below show a section of approximately 50 mm on a side.

The set of mineral samples presented in this work comprises five groups totalling 307 mineral samples. The diversity of grain size and mineral composition profiles can be seen in Fig. 1. These five sample groups have different origins. Only one of them (PORPHYRY) has been constructed by our research group. The other four sets were provided to us by industry partners in order to use them for research purposes and their characterisation was performed by third-party companies that are suppliers to the large-scale mining industry for mineral characterisation. Consequently, the response variables provided meet the standards used by the industry to model their deposits and plan their operations.

The acquisition and processing of the hyperspectral images have been done in-house. As a complement to the hypercubes, the database includes RGB images produced from the original hyperspectral image, applying the method explained in the section “Generation of reference RGB images”. In these images, the grain profile and texture of the minerals can be observed for each sample.

The hyperspectral images have been manually cropped to contain as much material and as little background as possible with the method explained in the section “Image cropping process”.

For each set of mineral samples, the following subsections present an overview and the characterisation methods with which the response variables were generated for use in training the learning models.

#### GEOMET - Spectra of geometallurgically characterised drill core mineral samples from a porphyry copper deposit

This dataset comprises 146 ore samples that were also metallurgically tested. The response variables provided were obtained from laboratory metallurgical tests on composite samples taken from 20-meter drill hole segments. The first four variables (Cu rec, Mo rec, pH, Lime cons) were obtained from unit cell Rougher Flotation tests; the fifth (WI) was obtained from a standard Bond Work Index test which is described in Wills, 2015, chapter 5^5^.

All samples have associated hyperspectral images. To work with these data, attention can be paid to the copper recovery factor and its variability expressed in spectral information. The variation in pH is also relevant. Soluble minerals such as alunite influence acidity, for example, if the pH is low, it may imply a higher lime consumption in the flotation process. Another parameter of interest is the Work Index (WI), an indicator of the grinding effort that must be made to crush the rock. A high WI may be related to the presence of potassium feldspar, while a low WI may indicate the presence of quartz.

The physical samples in this dataset look like fine powders of different shades of grey.

These samples were subjected to a closed cycle laboratory batch flotation test, with 500 g of mineral sample per cycle. In a batch flotation test, the cells are mechanically agitated with variable impeller rotation speeds and simulate the customer’s commercial model. Air is injected into the cell through a vertical tube surrounding the impeller shaft, the volume of which is controlled by a valve and by the impeller rotation speed. The air stream is separated into small bubbles that rise through the slurry to the surface, collecting particles from the collection zone into the froth phase^5^.

The closed loop test is performed to study interconnected stages of flotation with recirculation. It is more laborious than the open cycle test as it is composed of several cycles. It is a set of repetitive batch flotation tests performed in the laboratory, which experimentally simulate a continuous processing circuit. Each test is composed of interconnected cycles in such a way that the intermediate products generated in each of them are recirculated as feed to the next cycle. In this case, six cycles were performed.

From the execution of a test, intermediate and final products will be generated. Intermediate products are commonly tailings from some cleaning stage or impure concentrates that must be recirculated to a particular stage of the next cycle until the end of the test.

Once the closed cycle test was completed, the products were filtered, dried, weighed, and sent to the laboratory, where characterization was performed by AAS - Atomic Absorption Spectrography (Standard Test AAS023D). Characterization tests were also performed on the feed samples in order to calculate the reported copper and molybdenum recoveries. The acidity level (pH) reported was obtained on the liquid phase of the sixth cycle (steady state) and the lime consumption was reported as the amount of lime used during the process per mass unit.

#### PORPHYRY - Artificial mineral mixtures for a copper porphyry standards database

It is a set of mineral samples created by combining different rock types, each with high proportions of specific minerals. The samples were prepared to represent typical alteration and mineralisation characteristics common to porphyry copper deposits. The variability of the spatial distribution of mineral rock types influences ore processing. This dataset can then serve as a crude, non-analytical standard to measure the efficiency of classification and regression algorithms using some of the most common gangue mineral ratios for a porphyry copper system.

Samples were prepared manually by mixing different amounts of minerals, including quartz, biotite, kaolinite, specular haematite, pyrite, molybdenite, and magnetite. Fine (<30) and intermediate (6–30) grain-size materials were used. These mesh designations correspond to apertures of 3.36 mm and 0.595 mm respectively.

Twenty-eight samples were created and grouped into eight sets, which are described below.

- Group Q1 mixed Quartz and Biotite; Quartz, Molybdenite, and Biotite, in a fine size powder (<#30).
- Group Q2 mixed Quartz and kaolinite; Quartz, kaolinite and molybdenite, in a fine (<#30) size homogeneous white powder.
- Group Q3 mixed samples of group Q1 and specular hematite; group Q1, specular hematite and pyrite; group Q1, specular hematite, pyrite and chalcopyrite. Homogeneous grayish powder where it is possible to identify pyrite and specular hematite grains by eye, in a fine (<#30) size.
- Group Q3 mixed samples of group Q1 and specular hematite; group Q1, specular hematite and pyrite; group Q1, specular hematite, pyrite and chalcopyrite. Homogeneous grayish powder where it is possible to identify pyrite and specular hematite grains by eye, in a fine (<#30) size.
- Group Q4 mixed minerals of group Q1 and magnetite; group Q1, magnetite, (chalcopyrite, pyrite and bornite). Fine grayish powder where it is possible to identify mineral grains of the sulfides and oxides by eye, in a fine size (<#30).
- Group Q5 Samples of group Q2 mixed with hematite. Homogeneous reddish powder of fine size (<#30).
- Group Q6 Mixed biotite, quartz, magnetite and pyrite. A heterogeneous mixture of grain size approximately 3.5 mm in diameter where the associated mineral type is clearly identified by eye. Intermediate-size (#6-#30).
- Group Q7 Mixed quartz and pyrite; quartz, molybdenite, and pyrite. Fine white powder. Intermediate-size (#6-#30).
- Group Q8 Mixed quartz, kaolinite; quartz, kaolinite and hematite. Homogeneous reddish grains of approximately 3.5 mm in diameter. Intermediate-size (#6-#30).

The units for the response variables (minerals in the mixture in each case) are in % of the total mass of each sample. There is a tag containing total mass information for each sample in the response variables file provided (see the Data Records section above).

#### MINERAL1 - Monthly composites of mineral feed samples from the grinding-flotation process characterised by modal mineralogy analysis for a porphyry Cu-Mo deposit

This dataset corresponds to monthly composites of ore feed samples taken each day from three different process lines, destined for different milling plants in a copper mine. These composites are prepared for QEMSCAN modal mineralogical analysis and separated into three size fractions (coarse: −500/+150 μm, fine: −150/+3 μm and mixed, which are both fractions together, prior to particle size separation).

The response variables for this set of samples comes from a modal mineralogy analysis in units of weight percent and are intended for clustering exercises to obtain major geometallurgical units, and for supervised experiments with any subset of mineral counts.

The set of mineral samples comes from monthly composites (S1 to S12 for fines and coarse and S1 to S9 for blend) of daily samples from three different process lines (P1 to P3) of milling for the flotation process. In the provided files of the response variables, there are corresponding labels in the column ‘tags’. Each size fraction for an Sn sample corresponds to its own unique mineralogical characterisation of 33 variables, which include specific mineral species and some variables that gather sets of trace minerals, totalling 100 percent for each ordered process-sample-size set.

A relationship between mineralogy and grain profile can be established to determine the liberation factor of the minerals of interest (copper). In the coarser fraction, higher proportions of chalcopyrite are associated with potassic alteration and B-type veins. Lower values of chalcopyrite in this fraction are due to disseminated pyrite-chalcopyrite mineralisation associated with phyllic alteration, where the chalcopyrite is enclosed within the pyrite and not well liberated. In this case, higher copper concentrations are found in the finer fraction.

The samples are homogeneous grayish powders with different weights ranging from 4 grams to 47 grams, 94 physical samples in total. Size fractions are:

1. Coarse −500/+150 μm
2. Fine −150/+3 μm
3. Mixture

The mineralogical characterization of the samples was carried out using the Bulk Mineralogical Analysis - BME method with the QEMSCAN equipment, which is an abbreviation standing for quantitative evaluation of minerals by scanning electron microscopy, and a registered trademark owned by FEI Company since 2009. This system creates phase assemblage maps of a specimen surface scanned by a high-energy accelerated electron beam along a predefined raster scan pattern. Low-count energy-dispersive X-ray spectra (EDX) are generated and provide information on the elemental composition at each measurement point. The elemental composition in combination with back-scattered electron (BSE) brightness and x-ray count rate information is converted into mineral phases.

This is a rapid line-reading method used to identify the number and length of intersections with mineral species in each line. The electron beam is incident on the sample in a series of parallel lines, with measurements made at a regular spacing in each line and normally the spacing between the lines is greater than within each line so that each particle is measured only once. The data obtained with this process were used to determine modal abundance (Goodall *et al*., 2005)^6^ and (Gottlieb *et al*., 2000)^7^.

The units for the response variables provided are in weight percentage (wt %).

#### GEOCHEM - Feed ore samples for copper sulphide milling and flotation process characterised by X-ray fluorescence

The mineral samples used for this dataset come from the fine fraction sucked from the chutes of six different feeders of a large stockpile delivering ore to a SAG (Semi-Autogenous Gridding) mill, which is the first stage of a grinding and flotation plant of a porphyry copper deposit. The predominant mineral species are quartz and muscovite, corresponding to phyllic alteration. The samples are labelled with standardised X-ray fluorescence analysis (18 geochemical elements).

A total of 28 mineral samples were used. These were composed of a homogeneous fine fraction of grey powder with a weight varying between 11 and 361 grams and a coarser fraction with a wide range of grammages in all cases except samples 27 and 28 which only had the fine fraction.

Elemental results were obtained by using an Olympus Delta Premium portable X-ray fluorescence equipment (pXRF) in representative samples from each source. The original samples had 100% - 100# Tyler particle sizes (particles smaller than 150 microns). They were divided in a rotary cutter into homogeneous parts and representative parts were used for analysis. The analysis was performed in duplicate for each sample’s previous pulverization to obtain 100% – 75 μm according to ASTM E 11- 01 standard (ASTM International, 2001). The elemental quantification obtained was corrected using rocks standards previously characterized by chemical analysis and provided by ORE: Ore Research and Exploration Company.

Each element given by pXRF has a limit of quantitation (LOQ), which is calculated statistically from analysis of the results when the assay is performed at least in duplicate (Kadachi and Al-Eshaikh, 2012)^8^. If the quantity obtained by pXRF is greater than LOQ, then the results are considered adequate if it was properly corrected using known standards. It is relevant to mention that LOQ is different to limit of detection (LOD), which is related to the lowest analyte concentration that can be recognized in comparison to a blank sample that contains the analyte, while LOQ is the lowest concentration that the analyte can be detected meeting some predefined goals for bias and imprecision. For this reason, LOQ is higher or equal to LOD.

X-ray fluorescence raw data were corrected using known standards of rocks (OREAS 151b–153b and OREAS 501b–504b) (ORE, 2017) and the analyses were performed in triplicate, so the LOQ could be calculated as explained by Kadachi and Al-Eshaikh (2012)0^8^. From the results, it can be noticed that all element values can be used.

The units for the 18 measured chemical elements are percentages by weight (wt %). The sum of these percentages for each representative homogeneous sample generated was 35% on average.

#### MINERAL2 - Drill core samples from a porphyry copper ore deposit characterised by X-ray diffraction

The mineral samples are from composites of 3 meters of drill core from a porphyry copper deposit. Semi-quantitative mineralogical variables are obtained by standardised X-ray diffraction characterisation and are part of the data set used to build the long-term three-dimensional geometallurgical model of the deposit.

A total of 20 samples of fine homogeneous grey powder with a grammage varying between 80 grams and 186 grams were used.

A Bruker® D8 Endeavor 3KW Bruker® D8 Endeavor Diffractometer with *θ*/*θ* geometry goniometer was used for XRD characterization. Samples were scanned in an angular range from 3 to 70 degrees θ. Measurements were performed with 0.02 degrees theta of Step at a rate of 12 minutes per diffractogram, using a tube with 35 kV and 40 mA cobalt radiation. The detector of the equipment is an LYNXEYE XE (Silicon strip), with a 4° aperture, Bragg-Brentano geometry, and a constant temperature of 22 °C.

For the quantification of crystalline phases, the Rietveld^9^ refinement was applied. This method consists of a theoretical adjustment of the diffraction pattern by applying a model that includes structural and experimental factors. The quantification is performed with TOPAS software, after identifying the phases present by using EVA software. The detection limit of this technique is 1% by weight of the mineral, so any value below this is reported as a trace.

Semi-quantification of clays in fine fraction (<2 um) was based on the Mineral Intensity factor (MIF) methodology. The clay content obtained in the semi-quantification was normalized to 100% of the Total Rock, obtaining a single table with the total list of minerals (including clay species). For this normalization, only the clay species montmorillonite, montmorillonite-illite, vermiculite, chlorite-vermiculite, and chlorite-montmorillonite obtained from the semi-quantification are considered, because the species chlorite, illite, pyrophyllite, and kaolin group have already been quantified by the Rietveld method applied in the Total Rock analysis.

### General geological context of the presented mineral sample sets

The following paragraphs present a brief description of the geological context of the four deposits to which the mineral samples provided by our industry partners for research purposes belong. A description of the most relevant characteristics of a copper porphyry is also included, which serves as a reference and justification for the choice of the minerals that are part of the fifth set of samples presented in this work that was constructed by our group. It is important to mention that the geological descriptions presented are only intended to give a general idea of the sites of the deposits from which the samples come, but that we have not been authorised by our industry partners to give more detailed information, as they are extremely jealous of their data and have asked us to present the information in such a way that it remains completely anonymous.

#### GEOMET

For this dataset, drill core samples were taken from a porphyry copper deposit in the Neogene metallogenic belt of the central Chilean Andes. At least seven different types of tourmaline breccias occur in this deposit, which is characterised by their location, mineral composition of the matrices, types, and sizes of fragments, shapes, types, and degrees of mineralisation and alteration. Most breccias are usually monomictic with matrices of varying amounts of quartz, tourmaline, specular haematite, anhydrite, pyrite, chalcopyrite, bornite, molybdenite, sericite, chlorite and rock flour.

#### PORPHYRY

Porphyry copper deposits are characterised by alteration-mineralisation zonation that is described by a spatio-temporal conceptual model that has evolved from the original proposal by Lowell & Guilbert (1970)^10^ to Sillitoe (2010)^11^. These zonal patterns, in general, are centrally ordered from bottom to top by deep-rooted lateral chalcocitic alteration. This deep-rooted alteration is cut by a low-grade core of potassic alteration, which grades laterally and upwards to a halo of propylitic alteration. These early alterations are overlain and cut by upper-deepening chlorite-sericitic and sericitic (phyllic) alteration that grades upwards to late advanced argillic alteration that constitutes a broad and widespread lithocap (Sillitoe, 2010). The chalcocitic alteration is characterised by minerals such as albite/oligoclase, actinolite and magnetite with magnetite ± actinolite veinlets. The potassic core concentrates ore zones along its outer rim, surrounding a low-grade core with disseminated and micro-vein mineralisation, which grades into veins, stockworks and disseminations. Alteration assemblages consist of minerals such as biotite, potassium feldspar (K-feldspar), and quartz, with the main sulphide minerals being chalcopyrite-pyrite, chalcopyrite ± bornite, bornite ± digenite, and contemporaneous veins of biotite, potassium feldspar, quartz-biotite, sericite-potassium feldspar-andalusite-sulphides, quartz-sulphides ± magnetite, quartz-molybdenite ± pyrite ± chalcopyrite; groups Q1 and Q4 contain mineralogy characteristic of this sector. Chlorite-sericite alteration is imposed from top to bottom on the potassic core with chlorite, sericite (illite and hematite, and pyrite-chalcopyrite as main sulphides with chlorite ± sericite ± sulfide veins). The minerals added to group Q1, which are hematite, pyrite, and chalcopyrite are related to this alteration and form groups Q3 and Q6.

The chlorite-sericite alteration grades to quartz-sericite (phyllic) alteration, which totally or partially destroys the potassic and chlorite-sericite alterations. Mineralisation in this alteration zone consists mainly of pyrite ± chalcopyrite (pyrite-enargite ± tennantite, pyrite-bornite ± chalcocite, pyrite-sphalerite). Mineralisation occurs in quartz-pyrite veinlets ± other sulfides, stockworks, and dissemination, which is represented in group Q7. Late advanced argillic alteration is present at the top of porphyry copper systems, commonly constituting the so-called lithocap (Sillitoe, 2010)^11^, and is identified by the presence of residual vuggy quartz, alunite, pyrophyllite, dickite, kaolinite, some of these minerals can be identified in groups Q2, Q5, and Q8. Associated mineralisation consists of pyrite-enargite, pyrite-calcite, and pyrite-covellite in seams, stockworks and dissemination. The entire porphyry copper system is surrounded by a propylitic alteration halo, characterised by chlorite, epidote, albite and carbonates, with pyrite (±sphalerite, galena) as the main sulfide, mostly in seams.

#### MINERAL1

The samples come from a porphyry Cu-Mo deposit located in the central Andes of Chile, belonging to the Neogene metallogenic belt. It was formed by magmatic-hydrothermal processes associated with several episodes of felsic intrusions, which in turn are related to different stages of progressively superimposed hydrothermal alterations. A first sodic-calcitic stage is cut and followed by a potassic and propylitic stage extending from the core outwards and upwards. These early alterations and zones of mineralisation are overlain by phyllic and chlorite-sericite alteration from top to bottom. Late-phase phyllic alteration overlays all previous alteration phenomena. Most of the copper and molybdenum mineralisation occurs in veins, stockworks and in the matrix of hydrothermal breccias. Bornite, chalcopyrite and pyrite are the main sulphide minerals, occurring throughout different zones of the deposit.

#### GEOCHEM

These samples come from a porphyry copper deposit in the northern Andes of Chile. In this deposit, a zonal pattern of hydrothermal alteration and mineralisation occurs, centred on a porphyry stock. It is characterised by hydrothermal biotite, K-feldspar, quartz, anhydrite, and sulfides, predominantly chalcopyrite, bornite, pyrite and small amounts of molybdenite.

#### MINERAL2

The samples come from a porphyry copper deposit in northern Chile. The deposit is characterised by a superposition of hydrothermal alterations. The samples come from a supergene environment in transition to a zone of primary mineralisation, characterised by oxidised copper minerals at the surface, mainly atacamite and chrysocolla, and with the presence of minerals such as bornite, chalcopyrite and pyrite in deeper zones.

### Experimental Setting

**Fig. 2:**
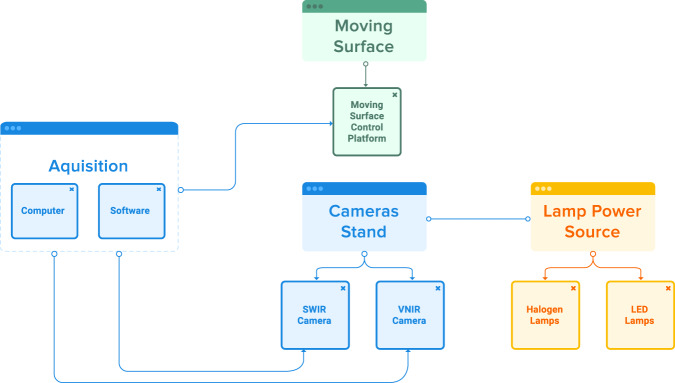
Acquisition system general schema.

**Fig. 3:**
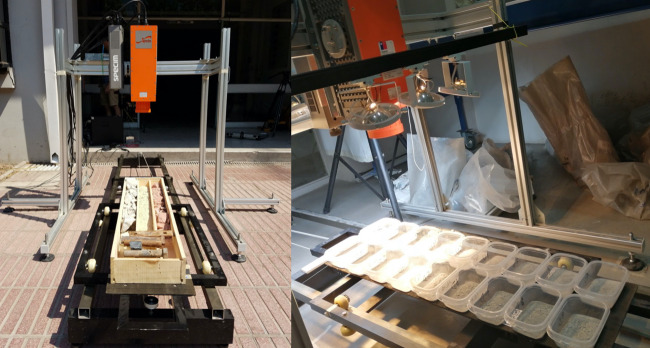
Experimental setup.

The experimental setting used to create the hyperspectral imaging database has the following components, as shown in Fig. 2: A mobile platform with its control device to move the samples under the lens of the hyperspectral cameras, a stand that holds the cameras and lamps in a firmly fixed position, two hyperspectral cameras, halogen and led lamps together with their DC power supplies, a computer including acquisition cards and proprietary acquisition software that interacts with the cameras and the mobile platform in a coordinated way. See Fig. 3.

#### Sample movement platform hardware structure and control device

The motion platform was designed and built to the specifications of a dolly rail for film cameras; therefore, it moves smoothly under the action of a precision stepper motor. The control platform is Arduino-based and connects to the acquisition computer via a USB serial port. The acquisition software allows for defining the speed of movement and controlling the start and stop sequences to synchronise with the camera acquisition process.

#### Illumination setting

The lighting configuration includes low-cost *AR*111 form factor tungsten halogen lamps and Philips 15 W 12 V 2700 K photo-led lamps (same *AR*111 form factor) to improve the visible range. The lamps are mounted on two illumination rails attached to the camera support structure: three halogen lamps pointing at the SWIR scan line and three halogen lamps and two LED lamps pointing at the VNIR scan line. The lamps have a beam angle of 20 degrees and are aimed at both scan lines to cover them evenly. However, some intensity variability is provided along the scan lines. Two DC power supplies are used to avoid flicker.

The hyperspectral images in this database are intended to incorporate the variability of environmental conditions, so the experimental setup is not isolated from natural ambient illumination (sunlight) that might enter the laboratory slightly.

#### SWIR camera

The SWIR spectral range is acquired with a SPECIM SWIR MCT camera, with a 9.2 × 6.9 *mm* (384 × 288 pixels) sensor. The spectrograph model is *N*25*E*22/3”. This configuration provides 288 spectral bands (with a spectral range of 906–2584 nm, and with a nominal spectral range of 1000–2500 nm). The nominal spectral resolution is 12*nm* with a nominal spatial curvature of 0.75*nm*. The numerical aperture is 2. The scan line has 384 pixels. High signal-to-noise ratio 900:1 (at maximum signal level). Spatial and spectral binning is allowed.

#### VNIR camera

The VNIR spectral range is acquired with a Specim VNIR sCMOS camera (Andor Zyla sCMOS), with a 13.83 × 6.29 *mm* (2128 × 968 pixels) sensor. The spectrograph model is *V*10*E*2/3”. This configuration provides 968 spectral bands (with a spectral range of 392–1008 nm, and with a nominal spectral range of 400–1000 nm). The nominal spectral resolution is 2.73*nm* with a nominal spatial curvature of 0.125*nm*. The numerical aperture is 2.4. The scan line is 2128 pixels. Spatial and spectral binning is allowed. Signal to noise ratio 170:1 (without binning) to 680:1 (with binning 8 × 2).

#### Computer characteristics

The computer used for the acquisition of the hyperspectral images is a Dell Precision T1600, with a Xeon E3–1200 processor and 32GB of RAM. All image processing steps have been performed using a Dell Precision 7910 with an Intel Xeon Dual Processor E5-2620 v4 with 3.0 GHz and 64GB - 2400 MHz of DDR4 RAM. Data was stored on a WD My Cloud Pro EX4100 6TB NAS in Raid 1 configuration.

#### Acquisition cards and protocols

Both cameras communicate with the acquisition equipment via dedicated PCI cards with Camera-Link protocol. These cards have their own independent drivers provided by the manufacturer.

#### Acquisition software

For image acquisition tasks, we have implemented a custom frame grabber developed in Python 3.6 with bindings for the Specim SDK. This application allows the visualisation and storage of the acquired images. It also includes an interface to control the motion platform.

### Hyperspectral images acquisition

Since the sensors are line scanners, to obtain a two-dimensional image of a sample, it must move under the field of view of the sensors, while the frame grabber acquires the information. A motorised tray controls the speed of sample displacement and is calculated to maintain an appropriate aspect ratio in the resulting image.

**Fig. 4:**
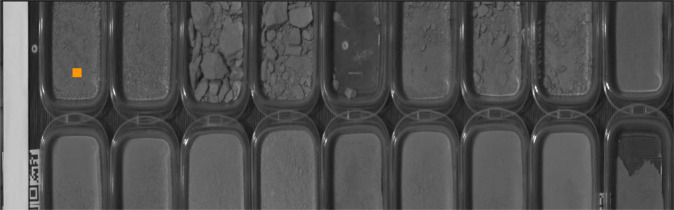
This image is monochromatic and was generated from the total energy of all the bands in each pixel of a VNIR image. The plastic containers are 130 by 100 internal size and the orange square is 5 by 5 pixels. On the left is the white band of the Optopolimer that we use as a white reference. The mineral samples for the spectral acquisition process are placed in these containers for each measurement.

The mineral samples are placed in such a way that a large part of them is exposed on their surface. Plastic containers or cardboard trays are used to sense each mineral sample. If the material is abundant, more than one image (measurement) is taken and each time the mineral is moved to expose another portion of it to the surface. Different sizes of plastic trays are selected depending on the mass of each sample. An example arrangement of ore samples is shown in Fig. 4. Here, each plastic tray contains one sample. As part of the pre-processing step, one crop is obtained for each mineral sample to present a separate image of each sample.

The acquisition system provides a set of parameters to be configured. Each hyperspectral camera allows defining the frame rate (1–400 Hz for the SWIR camera, 1–100 Hz for the VNIR camera), the integration time or exposure time range (0.1–20 ms for the SWIR camera, 8.1–100 ms for the full-frame VNIR camera), the spectral binning (1x–8x for both cameras) and the spatial binning (1x–8x for both cameras). In addition, in order to provide a dark reference for the hyperspectral reflectance data, a set of frames is acquired with the shutter closed. Finally, each image includes a strip across the width with a material that serves as a white reference.

**Fig. 5:**
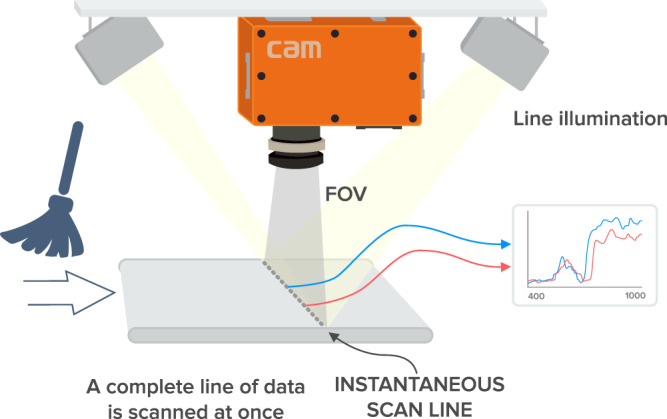
Schematic of push-broom concept; parts of this schema belongs to *SPECIM*^(*TM*)^ web page.

Samples in trays are placed on a sliding device that gently moves the samples under the focus of the line scanners. The camera lenses are placed 56 cm (SWIR) and 48 cm (VNIR) above the tray surface. The SWIR camera has a focal length of 22.5 mm and a FOV of 23 degrees. The VNIR camera has a focal length of 23 mm and a FOV of 34.3 degrees. The scan line covers 23 cm (VNIR) and 29 cm (SWIR) of displacement in width along the surface of the system. The displacement tray crosses the scan line and both the VNIR and SWIR cameras acquire their spectral bins. See Fig. 5.

The SWIR camera uses spatial binning 1, which provides a width of 384 pixels and the VNIR camera uses spatial binning 2 with one exception (see 0), which provides a width of 1064 pixels. For SWIR the pixel width is 0.6 mm and for VNIR the pixel width is 0.27 mm. The spectral binning is set to 1 for both cameras (maximum spectral resolution in both cameras) with one exception (see Table 1). The sampling frequency for both cameras is 50 Hz, the integration time is 4 ms for SWIR and 12.7 ms for VNIR, and the dolly speed is 40 mm/s.

**Table 1:** Spacial and Spectral Binning for Raw Images.

Data Set/Camera	SWIR		VNIR	
**GEOMET**	width: 384 px (binning 1)	bands: 268 (binning 1)	width: 532 px (binning 4)	bands: 471 (binning 2)
**PORPHYRY**	width: 384 px (binning 1)	bands: 268 (binning 1)	width: 1064 px (binning 2)	bands: 942 (binning 1)
**MINERAL1**	width: 384 px (binning 1)	bands: 268 (binning 1)	width: 1064 px (binning 2)	bands: 942 (binning 1)
**GEOCHEM**	width: 384 px (binning 1)	bands: 268 (binning 1)	width: 1064 px (binning 2)	bands: 942 (binning 1)
**MINERAL2**	width: 384 px (binning 1)	bands: 268 (binning 1)	width: 1064 px (binning 2)	bands: 942 (binning 1)

### Preprocess

#### Outliers correction process

A simple outlier correction process was carried out for each raw image acquired. The first objective was to replace some known dead bands in a series of pixels of the SWIR MCT sensor. This substitution was performed by cubic interpolation with five bands on each side of each dead band. The second objective was to detect saturated bands or bands of very low intensity due to spurious metallic or dark particles in the sample or some unintended reflective or absorbing background pixel. In these rare cases, the algorithm simply replaces the intensity value by the simple average of one band on each side. The threshold for detecting these anomalies was defined as 100 units of the digital number range (0 to 100 and 65255 to 65350).

#### Reflectance image generation

The acquisition process includes the application of a calibration function provided by the Specim SDK used in our proprietary acquisition software. This function also makes use of the calibration file provided for each camera.

To obtain a reflectance image from the acquired raw images, a flat field correction process is performed for each image. A very well-described example of this method, applied to a frame-based hyperspectral camera, is described in Kokkal *et al*. 2019. This correction process is based on the following equation:

1 $${L}_{f,p}=\frac{{R}_{f,p}}{{S}_{f,p}}$$

where *L*_*f, p*_ is the radiant source spectrum, *R*_*f, p*_ is the radiance spectrum captured by the sensor and *S*_*f, p*_ is the desired reflectance spectrum; all of them per frame and per pixel of the acquired hyperspectral cube.

In order to obtain *L*_*p*_, all images are acquired with a white reference material included in the scene, which is described in the next section, and which has a known reflectance spectrum provided by the manufacturer. This spectrum is a reflectance, so we call it *S*^*W*^, which has the same value for each pixel. The radiance of the white reference ${R}_{p}^{W}$ of each image is taken, for each pixel of a frame, and in addition a dark reference *D*_*p*_ is obtained, running the acquisition with the shutter closed once each time an image is acquired. Applying the flat field correction (Eq. 1) we have:

2 $${L}_{p}=\frac{{R}_{p}^{W}-{D}_{p}}{{S}^{W}}$$

where we subtract the dark noise produced in the sensor from the radiance of the white reference. The *L*_*p*_ obtained is the same for each frame of the image and is different for each pixel of a frame.

To obtain the reflectance of the whole image we use again the Flat Field Correction (Eq. 1), this time changing *L* by *S* and substituting the value *L* by the one obtained using the Eq. 2. Then,

3 $${S}_{f,p}^{I}=\frac{{R}_{f,p}^{I}-{D}_{p}}{{L}_{p}}$$

where ${S}_{f,p}^{I}$ is the reflectance spectrum for each pixel in each image frame, and ${R}_{f,p}^{I}$ is the radiance spectrum for each pixel in each image frame. *D*_*p*_ is the sensor dark noise obtained for each pixel in an image frame.

#### Numerical representation of reflectance

In this database, reflectance is presented as a number between 0 and 65535, although it is usually presented as a percentage or a number between 0 and 1 - stored as a floating point. The reason is that, in this case, the number of bytes required in the computer’s storage memory is considerably smaller. Namely, the smallest floating point precision used in today’s computers is 32 bits, while for integers it is 4 bits. To decrease the size of the images and maintain adequate precision, a 16-bit integer was used, where the stored value is the reflectance (as a number between 0 and 1) multiplied by the range allowed by the 16-bit integer, which is 65535. Using this form of storage we obtain a difference of 10^−5^ (0.001%) between two consecutive values, which is a good accuracy and the same one used by the sensor. This simple strategy reduces the file size by 50% (!).

#### White reference material characteristics

**Fig. 6:**
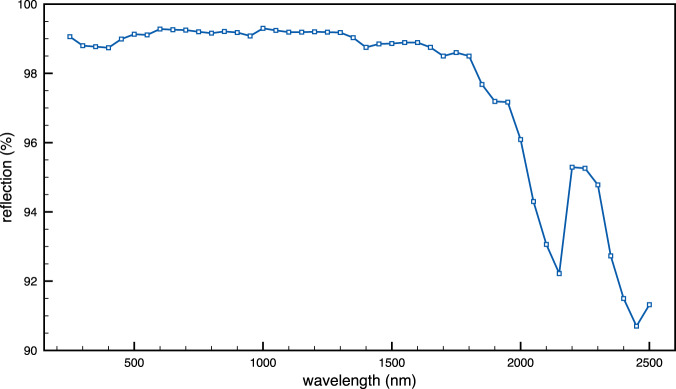
Reflection % in the VNIR-SWIR range for the white reference material (Optopolimer) used in our setup. Note that this graph provided by the manufacturer does not correspond to reflectance, but to reflection, as it is obtained by measuring the direct reflection of a real flat emission source.

A diffuse reflective material called Optopolymer® is used as a white reference material. According to the manufacturer, the diffusive optical reflective material Optopolymer® is a plastic that is used in numerous photometric and optical metrology applications, due to its high production quality, purity and highly diffusive reflectance behaviour. Some of the characteristics of this white reference material are wavelength range from 250 nm to 2,500 nm; reflectance >98.5% in the visible and 93% in the wavelength range of 250–2.500 nm; density 1.5*g*/*cm*3; operating temperature range −200^*circ*^ C to +260^*circ*^ C; hardness 30–40 Shore *D*; water-insoluble; high chemical resistance; non-flammable according to UL class V-0; high UV stability. The frequency response for the range of interest can be seen in Fig. 6 For this work a rectangular section of Optopolymer, with dimensions 40*cms* × 10 *cms* × 1 cms was used.

For each sample acquisition process, the reference target material is included in the scene, trying to cover the entire scan line of the cameras. It is placed at the same focal length as the samples, to ensure that the illumination observed on the reference target material is the same as that received on the samples under study.

#### VNIR-SWIR fusion process

To generate the complete range (400–2500 mn) spectra version for each mineral sample hyperspectral image a sensor fusion process was carried out. Both cameras are fixedly mounted, their scan lines are parallel arranged and the acquisition was performed simultaneously using both devices. There are only some alterations that are due to a change in focal length in samples or due to a loss of a frame by one of the grabbers during the acquisition process.

The scan lines of both cameras are different. Images were taken at the same time, but one scan line is displaced with respect to the other and they have a different number of pixels. It was assumed that the images have a non-empty visual intersection for the sensor fusion.

To obtain the transformation we used the sci-kit image processing Python library, which provides an automatic method to estimate transformations. Given the nature of the problem, we selected the projective transformation. This type of transformation requires 4 points of control, 4 in the source image (VNIR) and 4 in the destination image (SWIR). The projective transformation is widely used for change of perspective in computer vision because it can make corrections to the projection distortions changing the projection point from the actual point to the desired one; this method is described in detail in Hartley & Zisserman, 2004^12^. This problem also can be seen as a problem of distortions in the projection, because of the position of the cameras and their resolutions.

The mentioned transformation, which takes the VNIR image to the SWIR image coordinates was applied. The final fusion was done by concatenating the bands–and dropping some redundant bands. The resulting image has the SWIR dimensions.

#### RGB reference images generation

To generate RGB reference images from hyperspectral images, a hyperspectral image fusion approach based on bilateral filters was used. The idea is to fuse the whole set of bands to one monochromatic band in such a way as to highlight the edges in the final image to make it look sharp and focused. A brief summary of this method, detailed in Chaudhuri 2013^13^ pages 43–46, follows.

The general expression for a bilateral filter *I*^*BF*^ for a 2-D image is given by Eq. 4. It is pixel-based and takes into account the surrounding pixels $(\widetilde{x},\widetilde{y})$ in the spacial (Eq. 5) and range (Eq. 6) dimensions using Gaussian kernels. Equation 7 shows the normalization factor *W* calculation.

4 $${I}^{BF}\left(x,y\right)=\frac{1}{W\left(x,y\right)}\sum _{\widetilde{x}}\sum _{\widetilde{y}}\left\{{G}_{\sigma S}\left(x-\widetilde{x},y-\widetilde{y}\right){G}_{\sigma R}\left(I\left(x,y\right)-I\left(\widetilde{x},\widetilde{y}\right)\right)I\left(\widetilde{x},\widetilde{y}\right)\right\}$$

5 $${G}_{\sigma S}\left(x,y\right)=\exp \left(-\frac{{x}^{2}+{y}^{2}}{2{\sigma }_{S}^{2}}\right)$$

6 $${G}_{\sigma R}\left(\zeta \right)=\exp \left(-\frac{{\zeta }^{2}}{2{\sigma }_{S}^{2}}\right)$$

7 $$W(x,y)=\sum _{\widetilde{x}}\sum _{\widetilde{y}}\left\{{G}_{\sigma S}\left(x-\widetilde{x},y-\widetilde{y}\right){G}_{\sigma R}\left(I\left(x,y\right)-I\left(\widetilde{x},\widetilde{y}\right)\right)\right\}$$

It is basically designed to preserve edges, so when applied to a hyperspectral fusion process, it allows highlighting areas of a hyperspectral image where sudden and weak spectral features occur as well as edges in the spatial dimension.

Thus, in this case, the *hyperspectral bilateral filter* (Eq. 8) is a weighted sum of the *I*_*k*_ which are all the monochromatic images (or bands in the spatial dimension) of the original hyperspectral cube to be fused. The weights *w*_*k*_ are calculated as normalised subtractions of the filtered bands from the original ones, as shown in Eq. 9.

8 $$F\left(x,y\right)=\mathop{\sum }\limits_{k=1}^{K}{w}_{k}\left(x,y\right){I}_{k}\left(x,y\right)$$

9 $${w}_{k}\left(x,y\right)=\frac{| {I}_{k}\left(x,y\right)-{I}_{k}^{BF}\left(x,y\right)| +C}{{\sum }_{k=1}^{K}(| {I}_{k}\left(x,y\right)-{I}_{k}^{BF}\left(x,y\right)| +C)}$$

where *K* is number of spectral bands and *C* is constant. In our case, *C*=0.1.

#### Image cropping process and final images storage

Finally, once the pre-processed and fused hyperspectral images were obtained, regions of interest were selected from the images. As the mineral samples were arranged in trays, and due to the field of view of the cameras, there were many regions of the images that were not useful (the surface on which they were mounted, the sample containers, the reference points, etc.).

The selection of the regions of interest was done manually by defining rectangles containing only the hyperspectral pixels of each mineral sample. As the edges of the mineral surface were not perfectly straight, the selected rectangular sections left out some mineral-containing pixels, with the advantage of not having any pixels without mineral included.

Once these rectangles were defined, new HDF5 files containing the hyperspectral pixels, along with the associated spectral range of each sample, were created. Each sample has at least three hyperspectral cubes (which is called ‘one take’), a full spatial resolution VNIR image, and a paired set (created by the fusion process) of a SWIR and a low-resolution VNIR image. In some cases, a sample can count with more than ‘one take’. This corresponds to samples that have less mass and that are registered more than once, exposing different at their surface each time, just to provide more pixel variability for each sample.

## Data Records

The data set is published on Figshare^14^ as a collection with 5 different data records. The data included is separated according to:

- GEOMET (GMET) contains 146 samples with 1 measurements each. Contains 5 geometallurgical variables.
- PORPHYRY (POR) contains 28 samples with 2 measurements each. It has 10 variables available and particle sizes: “coarse” and “fine”.
- MINERAL1 (M1) contains 99 samples with 1 measurements each. Particle size “fine” contains 12 samples for each of the three processes, particle size “coarse” contains 12 samples per process, and particle size “mixed” contains 9 samples per process. Additionally, there are 33 variables available.
- GEOCHEM (GCH) contains 28 samples with 4 measurements each. Except for three samples (GCH-0023, GCH-0027, and GCH-0028) with 2 measurements each. It has 25 variables available for each sample, and particle sizes: “coarse” and “fine”.
- MINERAL2 (M2) contains 20 samples with 1 measurements each. It has 25 variables available.

A data record is composed of many samples. Each data record consists of a zip file that includes all samples as subfolders, where #### is the ID of the sample. The data records also contain the wavelengths of the samples (provided in the file wavelengths.json), which is the same for all data records. The summary for each data record, its variables, and its metadata are available in spreadsheet files (as supplemental material) GEOMET.xlsx, PORPHYRY.xlsx, MINERAL1.xlsx, GEOCHEM.xlsx, and MINERAL2.xlsx. Table 2 shows the metadata for the first 6 samples of database GEOMET, as an example of the data contained on these tables.

**Table 2:** Preview of initial 6 samples on data record GEOMET including metadata obtained from JSON files.

sample_id	Cu rec	Mo rec	PH	Lime cons	WI	CROP	tags	kind	*image_dims@width*	*image_dims@height*	*real_dims@width*	*real_dims@height*
**GMET-0001**	85.5	63.5	7.7	0.12	14.5	1		swir_low	145	72	89.9	58.7
**GMET-0001**	85.5	63.5	7.7	0.12	14.5	1		vnir_low	145	72	89.9	58.7
**GMET-0001**	85.5	63.5	7.7	0.12	14.5	1		vnir_high	160	74	44.3	60.3
**GMET-0002**	86.6	43.8	8.7	0.16	16	1		swir_low	100	87	62	70.9
**GMET-0002**	86.6	43.8	8.7	0.16	16	1		vnir_low	100	87	62	70.9
**GMET-0002**	86.6	43.8	8.7	0.16	16	1		vnir_high	111	88	30.7	71.7
**GMET-0003**	87.8	9	8.9	0.11	15.7	1		swir_low	131	90	81.2	73.4
**GMET-0003**	87.8	9	8.9	0.11	15.7	1		vnir_low	131	90	81.2	73.4
**GMET-0003**	87.8	9	8.9	0.11	15.7	1		vnir_high	145	91	40.1	74.2
**GMET-0004**	84.6	49.3	7.2	0.17	12.1	1		swir_low	130	89	80.6	72.5
**GMET-0004**	84.6	49.3	7.2	0.17	12.1	1		vnir_low	130	89	80.6	72.5
**GMET-0004**	84.6	49.3	7.2	0.17	12.1	1		vnir_high	144	90	39.9	73.4
**GMET-0005**	89.9	57.3	8.4	0.11	13.5	1		swir_low	131	87	81.2	70.9
**GMET-0005**	89.9	57.3	8.4	0.11	13.5	1		vnir_low	131	87	81.2	70.9
**GMET-0005**	89.9	57.3	8.4	0.11	13.5	1		vnir_high	145	88	40.1	71.7
**GMET-0006**	91	57.6	7.2	0.11	11.8	1		swir_low	134	82	83.1	66.8
**GMET-0006**	91	57.6	7.2	0.11	11.8	1		vnir_low	134	82	83.1	66.8
**GMET-0006**	91	57.6	7.2	0.11	11.8	1		vnir_high	149	84	41.2	68.5

**Fig. 7:**
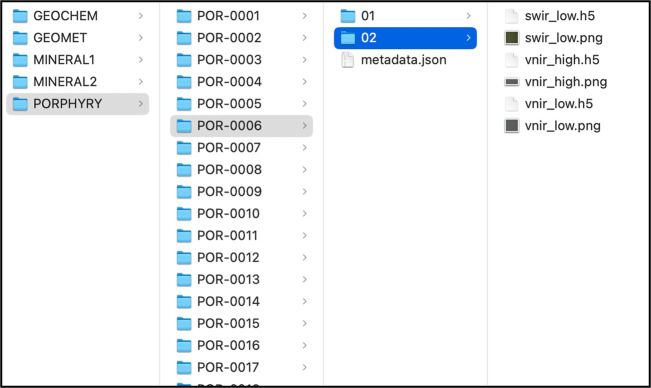
File structure for PORPHYRY data record listing the sample POR-0006 and measurement 02.

**Fig. 8:**
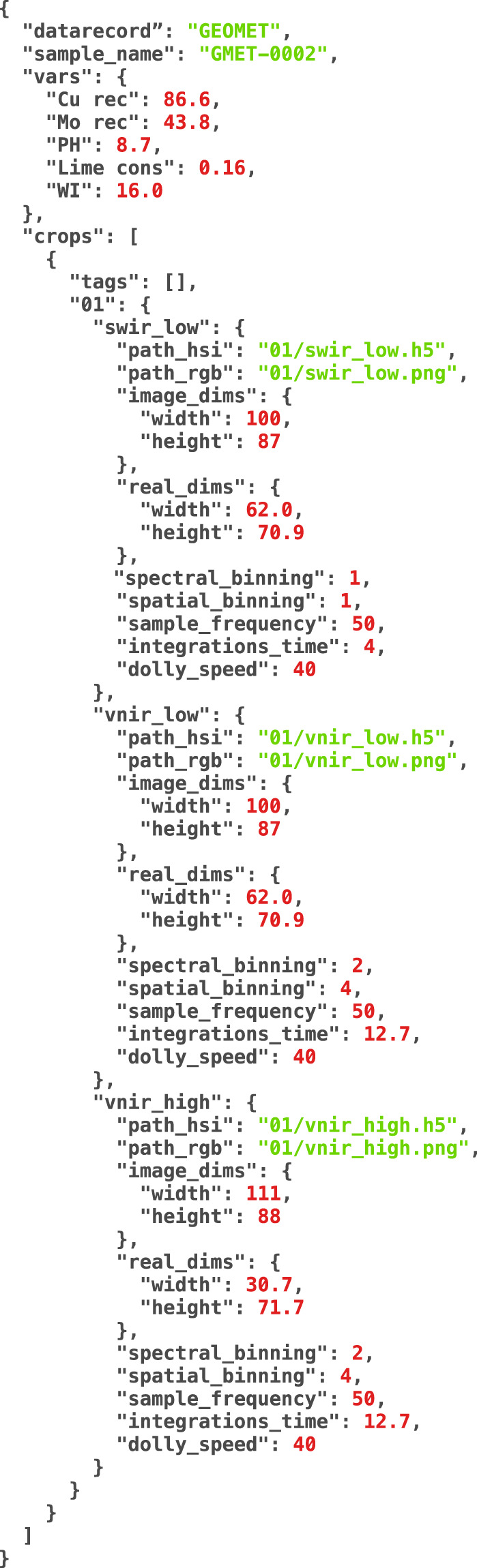
JSON metadata file for GEOMET data record listing the sample GMET-0002.

Each data record has a short name %%% (GMT, POR, M1, GCH, and M2) and each subfolder sample contains a JSON metadata file: metadata.json. It also contains the data of one measurement, at possibly four different shots (or crops) organized by folders. Each measurement has three hyperspectral images: SWIR LOW, VNIR LOW, and VNIR HIGH, and three PNG files according to each hyperspectral data file. The files within each folder are named as follows (#### is the sample ID, @ the number of the measurement):



The file structure for some samples on data record PORPHYRY is shown in Fig. 7. Here, the sample POR-0006 is highlighted, and as explained before, the folder contains a metadata.json file, and in this case, it contains only two measurements. The measurement 02 is shown where 6 files are listed: 3 PNG and 3 HDF5. Additionally, in Fig. 8 is displayed the content of the file metadata.json belonging to sample GMET-0002. For each sample, the JSON file contains the name of the data record, the sample name, the variables for the sample, and the specific data for each measurement, such as paths for hyperspectral files or PNG images, and their sizes which are useful to calculate the pixel size, based on the pixels from the image captured and the real size of the sample.

## Technical Validation

Each data set has been tested on different experiments, both for regression and classification, using as a base the proposed pipeline by Egaña *et al*.^15^.

**GEOMET** data record was extensively validated in Egaña *et al*.^15^. by testing it on a hierarchical regression pipeline, which consists of a clustering of stochastically selected patches of an image, a dimensionality reduction scheme for the preserved information, and a hierarchical regression model on the clusters. A random selection of 120 samples was made from the 146 available for the training process. For each sample, the five geometallurgical variables were measured. Then, 50 patches were obtained from each sample, representing a small section of 10 × 10 pixels in each image. In addition, each segment shares the same metallurgical measurement. The tests were performed on 21 samples that were left out of the forming process; for each sample in this test data set, 30 patches were created. From the prediction pipeline results, a set of histograms was generated showing the experimental distribution of the estimated geometallurgical variables. Here, the mean value of these distributions is considered as the final estimate to be compared with the measured values, through the mean absolute error (MAE) and root mean square error (RMSE). The Table 3 summarises the results, showing errors on average less than 5%.

**Table 3:** Performance of the pipeline with clustering and dimensionality reduction applied to GEOMET data record.

	RECCU	RECMO	PH	CONSCAL	WI
Dynamic Range	24.799	85.500	4.799	0.800	10.600
MAE	0.621	2.398	0.153	0.066	0.350
RMSE	0.853	3.400	0.189	0.071	0.441

**PORPHYRY** was validated by measuring accuracy in a classification experiment, in which a hierarchical classification pipeline was applied with the Random Forest classifier algorithm. The data record has eight categories, and each group has samples with variations in their mineral compositions. The experiment was run using rounds of exclusion. An accuracy of 79% was achieved using the 8 classes provided in the data record. The experiment was also interesting in that those samples that were classified into categories different from the origin were left with mineral compositions in which the most abundant minerals were the same, but the minority minerals changed, apparently not sufficiently for the sensitivity of the classification model.

**MINERAL1** was tested in multiple experiments with regression methods applied to different spectral sub-ranges for the same modal mineralogical response variables. The experiment used the aforementioned hierarchical method, where, after clustering the spots, a regression was trained for each cluster using the ordinary least squares regression algorithm. Training and testing rounds were performed for the three processes (P1, P2, and P3) and two size fractions (coarse and fine) as leave-one-out. Table 4 summarises the MAE in estimated values for 22 response variables out of the 33 available in the dataset.

**Table 4:** Mean Absolute Error on estimated values for MINERAL1 data record.

	P1		P2		P3	
	fine	coarse	fine	coarse	fine	coarse
**Chalcopyrite**	0.260	0.122	0.086	0.107	0.023	0.137
**Bornite**	0.133	0.039	0.035	0.035	0.006	0.014
**Chalcosite/Digenite**	0.009	0.008	0.011	0.005	0.064	0.010
**Covelite**	0.009	0.002	0.008	0.001	0.007	0.002
**Enargite/Tennantite**	0.004	0.005	0.003	0.003	0.011	0.005
**Tetrahedrite**	0.000	0.000	0.000	0.000	0.003	0.000
**Native Cu**	0.000	0.000	0.000	0.000	0.001	0.000
**Fe-Oxide(Cu)**	0.003	0.002	0.003	0.001	0.012	0.002
**Others Minerals Cu**	0.001	0.000	0.004	0.008	0.001	0.001
**Pyrite**	0.340	0.428	0.211	0.129	0.301	0.330
**Molybdenite**	0.013	0.015	0.012	0.009	0.009	0.005
**Fe Oxides**	0.132	0.088	0.018	0.073	0.040	0.014
**Quartz**	0.571	0.436	0.515	0.584	0.372	0.384
**K-Feldspar**	0.841	0.552	0.291	0.555	0.241	0.280
**Oligoclasa_An20**	0.367	0.200	0.781	0.771	0.720	1.074
**Muscovite/Sericite**	0.787	0.444	0.495	0.622	1.172	1.372
**Kaolinite**	0.049	0.096	0.039	0.075	0.040	0.192
**Biotite**	0.243	0.272	0.091	0.229	0.174	0.213
**Chlorite**	0.390	0.207	0.100	0.298	0.280	0.369
**Clay Cu**	0.001	0.001	0.001	0.003	0.001	0.004
**Fe-Clays**	0.040	0.047	0.049	0.091	0.039	0.017
**Anhydrite/Gypsum**	0.347	0.227	0.601	0.227	0.179	0.223

**GEOCHEM** data record was validated using the same method as in the previous cases, this time focusing on analysing the effect of reducing the number of bands and the spectral range of the cameras. To obtain the estimation error statistics for each response variable, leave-one-out rounds were performed. The probabilistic clustering and the ordinary least squares regression algorithm were applied for the three available data types: (i) FULL sensor, using concatenated VNIR and low SWIR bands, for which 1210 bands per spectral pixel are considered for the spectrum between 400 nm and 2500 nm; (ii) SWIR sensor, analysis using only the bands between 1000 nm and 2500 nm, where 268 bands are considered; (iii) lower cost Specim FX10 camera emulation by reducing bands in the 400 nm to 1000 nm range using 224 bands. Table 5 shows the mean absolute percentage error for 16 of the 28 available response variables. The advantage of using the FULL spectrum is evident for elements such as Ca, Zn, As or Pb, where the results are negatively affected by reducing the sensor spectral range.

**Table 5:** Mean Absolute Percentage Error of some XRF variables on GEOCHEM data record.

Element	% Full	% SWIR	% FX10
**Al**	0.021	2.476	2.260
**Si**	0.136	1.746	2.115
**P**	0.181	3.909	4.168
**S**	0.168	6.792	9.308
**K**	0.080	2.448	3.205
**Ca**	0.022	7.550	19.704
**Ti**	0.213	6.895	6.768
**Fe**	0.152	4.692	5.972
**Cu**	0.270	5.787	5.291
**Zn**	4.268	35.689	36.414
**As**	0.172	14.646	19.691
**Rb**	0.105	3.003	3.392
**Sr**	0.472	7.936	10.665
**Zr**	0.786	7.254	7.522
**Mo**	0.431	16.261	15.397
**Pb**	4.212	45.021	47.899

**MINERAL2** was validated by the same method as in the previous cases, using regression in a leave-one-out scheme. The experiment, using the pipeline with a hierarchical regression, was used to predict the Quartz value using all available hyperspectral information, using concatenated VNIR and low SWIR bands. After probabilistic clustering, the partial least squares regression algorithm was applied. Table 6 shows the results of each leave-one-out run, where the training operating error (mean absolute error divided by the dynamic range of the data) corresponds to 18.119%, and the mean absolute percentage error is 9.99%, with a lower error of 0,108% and an upper error of 33.285%.

**Table 6:** Real values, estimated values, and relative percentage errors for Quartz element using data record MINERAL2.

Sample ID	Real Value	Estimated Value	Relative Error [%]
**M2-0001**	56.640	52.356	7.563
**M2-0002**	54.806	56.294	2.715
**M2-0003**	44.329	59.084	33.285
**M2-0004**	56.831	60.200	5.928
**M2-0005**	62.739	51.834	17.381
**M2-0006**	48.255	57.950	20.090
**M2-0007**	51.670	50.638	1.998
**M2-0008**	58.468	60.230	3.013
**M2-0009**	48.876	52.446	7.304
**M2-0010**	60.240	53.809	10.676
**M2-0011**	52.126	47.366	9.131
**M2-0012**	53.782	56.611	5.260
**M2-0013**	41.022	44.360	8.136
**M2-0014**	56.009	62.587	11.745
**M2-0015**	57.393	52.522	8.488
**M2-0016**	60.486	54.639	9.666
**M2-0017**	59.927	59.862	0.108
**M2-0018**	54.394	60.483	11.194
**M2-0019**	55.769	60.864	9.137
**M2-0020**	71.084	58.905	17.133

## Usage Notes

There are four file types present in each data record: XLSX, JSON, PNG and HDF5. The first one summarises the information related to the samples in the data record. The second is used to describe each sample, its characterisation variables (mineralogy, X-ray diffraction values, geometallurgical assays, among others), the image size (width and height) and the paths containing the hyperspectral and RGB capture available for the current sample. The third is an RGB representation of each measurement. The latter is presented in at least three types: SWIR low, VNIR high and VNIR low, where low and high represent the resolution of the image.

In the following paragraph, an example code is provided to load any of the above.h5 files in Python. The hyperspectral image data is loaded from the HDF5 file. The first two dimensions are used to store the pixels, and the third dimension is used for the spectral information of each pixel. The example performs a cut in band 42.

- Python code (requires h5py and matplotlib.pyplot):

sample_file = h5py.File(‘vnir_high.h5’, ‘r’)sample_data = sample_file[‘/hsi_data’]plt.imshow(sample_data[:,:,42], cmap = ‘gray’)plt.plot(sample_data[20,300,:])


**Fig. 9:**
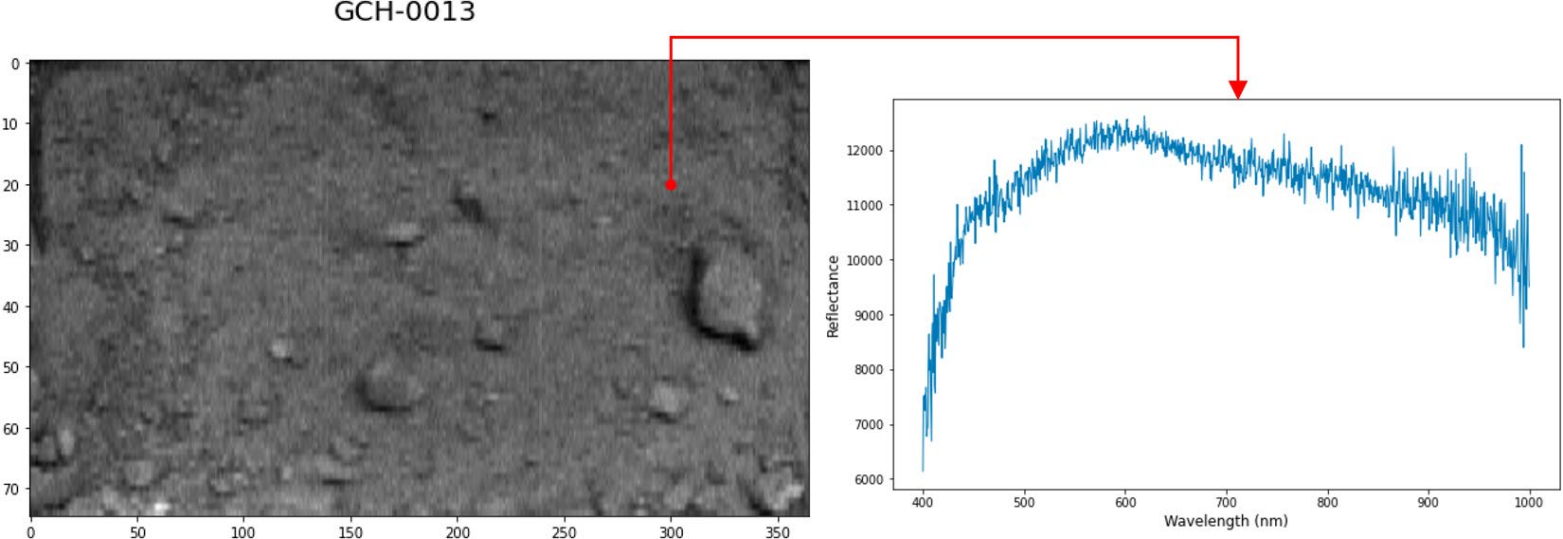
Grayscale image representation for GEOCHEM data record showing the sample GCH-0013 and the VNIR hyperspectral data for pixel (300, 20).

From the code shown, the resulting images are shown in Fig. 9 for the GEOCHEM data record and the sample GCH-0013. A red highlight is shown in Fig. 9 to indicate the pixel (20,300), which was used to obtain the spectral signature shown on the right.

As indicated in the Code Availability section, a Python library was developed to help perform simple operations on the dataset. The following subsections show some examples of use based on the mentioned Python module.

### Reconstruction of provided spreadsheets

Using only the data provided in a data record, it is possible to reconstruct a spreadsheet that summarises the information related to the sample, such as its variables, and the metadata pertaining to each crop or measurement available in the data record. For example, in the following code listing, the spreadsheet associated with the MINERAL2 data record is recreated. Note that the dataset path corresponds to the path where the data records were extracted.

- Python code using module pandas and the provided module hidsag:

import hidsagimport pandas as pddataset_path = ‘path/to/HIDSAG’hidsag.set_dataset(“MINERAL2”)samples_list = hidsag.get_samples_list(dataset_path)df_m2 = pd.DataFrame(samples_list)df_m2.set_index(‘sample_id’, inplace = True)df_m2.to_excel(‘MINERAL2.xlsx’)


### Filtering by metadata

The following code allows selecting a subset of samples by filtering by criteria according to the available metadata. For this example, the data record MINERAL1 will be used, being more interesting to select cases based on specific processes and particle size. The following code will select samples belonging to Process P2 and particle size ‘coarse’. A second filter will require samples from monthly composites S1, S2 or S4. The results are Pandas DataFrame with the MINERAL1 samples filtered on the variables filtered1_m1 y filtered2_m1.

- Python code using module pandas:

import pandas as pddf_m1 = pd.read_excel(‘MINERAL1.xlsx’)filtered1_m1 = df_m1[df_m1.tags.str.contains(r’^(? = .*P2)(? = .*coarse)’)]filtered2_m1 = df_m1[df_m1.tags.str.contains(‘S1|S2|S4’)]


### Exploring a single sample

The following code obtains a single sample from a data record and performs a simple listing of the available information regarding its metadata. From the data record GEOCHEM will list the variables associated with the sample, explore the number of available measurements or crops, obtain the associated metadata for a specific hyperspectral image (the available options follow the file names specified in the Data Records section: swir_low, vnir_low, and vnir_high), and select a specific hyperspectral image (vnir_low) to extract numerical data to finally plot band 42.

Python code using the provided module hidsag and matplotlib.pyplot:


import hidsagimport matplotlib.pyplot as pltdataset_path = ‘path/to/HIDSAG’hidsag.set_dataset(“GEOCHEM”)sample0011 = hidsag.get_sample(‘GCH-0011’)variables = sample0011.get_variables()crop = sample0011.list_crops()sample_metadata = sample0011.get_metadata(‘01’, ‘swir_low’)sample_vnir_low = sample0011.get_data(‘03’, ‘vnir_low’)plt.imshow(sample_vnir_low[:,:,42], cmap = ‘gray’, aspect = ‘auto’)


## Data Availability

The data records can be used straightforwardly as single files or as a set. A step-by-step example to extract the numerical data from each HDF5 file, along with a library developed for this purpose is available on GitHub: https://github.com/alges/hidsag. Along with the library, a Python notebook with sample usage is included containing: 1. The import of a data record from a path 2. The selection of a single sample 3. Slicing of a specific band and position for numerical data extraction 4. Visualization of the resulting slice 5. The list of each sample on the data record, its associated variables and metadata

## References

[1] Boisvert J, Rossi M, Ehrig K, Deutsch CV (2013). Geometallurgical modeling at olympic dam mine, south australia. Mathematical Geosciences.

[2] Fasnacht L, Vogt M-L, Renard P, Brunner P (2019). A 2d hyperspectral library of mineral reflectance, from 900 to 2500 nm. Scientific Data.

[3] Vapnik, V. N. An overview of statistical learning theory. *IEEE Transactions on Neural Networks* **10** (1999). 10.1109/72.788640 18252602

[4] Hastie, T., Tibshirani, R. & Friedman, J. *The Elements of Statistical Learning: Data Mining, Inference, and Prediction. Second Edition* (Springer, London, 2009).

[5] Wills, B. A. & Finch, J. *Wills’ Mineral Processing Technology, 8th Edition* (Elsevier, 2015).

[6] Goodall W, Scales P, Butcher A (2005). The use of qemscan and diagnostic leaching in the characterisation of visible gold in complex ores. Minerals Engineering.

[7] Gottlieb P (2000). Using quantitative electron microscopy for process mineralogy applications. JOM - Journal of the Minerals, Metals and Materials Society.

[8] Kadachi A, Al-Eshaikh M (2012). Limits of detection in xrf spectroscopy. X-Ray Spectrom.

[9] Rietveld HM (1969). A profile refinement method for nuclear and magnetic structures. Journal of Applied Crystallography.

[10] Lowell DJ, Guilbert JM (1970). Lateral and vertical alteration-mineralization zoning in porphyry ore deposits. Economic Geology.

[11] Sillitoe RH (2010). Porphyry copper systems. Economic geology.

[12] Hartley, R. I. & Zisserman, A. *Multiple View Geometry in Computer Vision*, second edn (Cambridge University Press, ISBN: 0521540518, 2004).

[13] Chaudhuri, S. & Kotwal, K. *Hyperspectral image fusion* (Springer, 2013).

[14] Ehrenfeld A (2022). Figshare.

[15] Egaña, Á. F. *et al*. A robust stochastic approach to mineral hyperspectral analysis for geometallurgy. *Minerals* **10**10.3390/min10121139 (2020).

